# Exploring economic empowerment and gender issues in Lesotho’s Child Grants Programme: a qualitative study

**DOI:** 10.1093/heapol/czad009

**Published:** 2023-02-08

**Authors:** Elodie Besnier, Thandie Hlabana, Virginia Kotzias, Kathryn Beck, Celine Sieu, Kimanzi Muthengi

**Affiliations:** Centre for Global Health Inequalities Research (CHAIN), Department of Sociology and Political Science, Norwegian University of Science and Technology (NTNU), PO Box 8900, Torgarden, Trondheim 7491, Norway; Department of Public Health and Nursing, Faculty of Medicine and Health Sciences, NTNU, PO Box 8900, Torgarden, Trondheim 7491, Norway; National University of Lesotho, P.O. Roma 180, Roma, Lesotho; School of Environmental Sciences, University of Hull, Cottingham Rd., Hull HU6 7RX, UK; Centre for Global Health Inequalities Research (CHAIN), Department of Sociology and Political Science, Norwegian University of Science and Technology (NTNU), PO Box 8900, Torgarden, Trondheim 7491, Norway; Centre for Global Health Inequalities Research (CHAIN), Department of Sociology and Political Science, Norwegian University of Science and Technology (NTNU), PO Box 8900, Torgarden, Trondheim 7491, Norway; Centre for Fertility and Health, Norwegian Institute of Public Health, P.O. box 222, Skøyen, Oslo N-0213, Norway; UNICEF Lesotho Country Office, 13 UN Road, UN House, Maseru, Lesotho; UNICEF Lesotho Country Office, 13 UN Road, UN House, Maseru, Lesotho

**Keywords:** Cash transfer, Lesotho, economic empowerment, social protection, gender, women’s empowerment

## Abstract

Cash transfers (CTs) have been increasingly used in low- and middle-income countries as a poverty reduction and social protection tool. Despite their potential for empowering vulnerable groups (especially women), the evidence for such outcomes remains unclear. Additionally, little is known about how this broad concept fits into and is perceived in such programmes. For example, Lesotho’s Child Grants Programme (CGP) is an unconditional CT targeting poor and vulnerable households with children. The CGP has been presented as one of the Lesotho’s flagship programmes in developing the country’s social safety net system. Using the CGP’s early phases as a case study, this research aims to capture how programme stakeholders understood and operationalized the concept of economic empowerment (especially women’s) in Lesotho’s CGP. The qualitative analysis relied on the triangulation of information from a review of programme documents and semi-structured key informant interviews with programme stakeholders. First, the programme documents were coded deductively, while the interview transcripts were coded inductively, and then both materials were analysed thematically. Finally, differences or disagreements within each theme were explored individually according to the programme’s chronology, the stakeholders’ affiliation and their role in the CGP. The complexity of economic empowerment was reflected in the diversity of definitions found in the desk review and interviews. Economic empowerment was primarily understood as improving access to economic resources and opportunities and, less so, as agency and social and economic inclusion. There were stronger disagreements on other definitions as they seemed to be a terminology primarily used by specific stakeholders. This diversity of definitions impacted how these concepts were integrated into the programme, with particular gaps between the strategic vision and operational units as well as between the role this concept was perceived to play and the effects evaluated so far.

Key messages

- The majority of sources and informants identified more than one dimension in their definitions of economic empowerment and women’s empowerment, thus illustrating the complexity of these concepts as applied to the CGP.
- Economic empowerment defined as access to economic resources and opportunities (for beneficiaries as a whole or for women in particular) was the most prominent and integrated dimension of economic empowerment identified by stakeholders across the programme.
- There were discrepancies and disagreements in the operationalization of these different concepts, particularly for their least agreed-upon definitions.
- The operationalization gaps identified in this study highlight how different empowerment processes may conflict with one another (affecting the impacts of the programme) and highlight systematic divisions, particularly between the strategic and operational levels of the programme.

## Introduction

Cash transfers (CTs)—non-contributory monetary transfers to individuals or households—have been increasingly used in low- and middle-income countries (UNICEF-ESARO, Transfer Project, 2015; Bastagli *et al.*, 2016). CTs have been associated with several human development outcomes, including empowerment, especially women’s and girls’ empowerment outcomes. However, their impact varies according to the indicator considered, the CT’s specificities or the context in which it is implemented (Bastagli *et al.*, 2016; Bonilla *et al.*, 2017; Peterman *et al.*, 2021). Lesotho’s Child Grants programme (CGP) started in 2009 is an unconditional CT targeting poor and vulnerable rural households with children. Its primary objective is to improve the living standards of orphans and vulnerable children (OVCs) to reduce malnutrition, improve health status and increase school enrolment (Pellerano *et al.*, 2014). Effectiveness and impact evaluations have explored the economic, livelihood, food security, child well-being and education effects of the CGP, as well as the contribution of selected design features, particularly as part of the Transfer Project (The Transfer Project, 2022). The 2014 CGP evaluation found promising effects amongst beneficiaries regarding selected economic and child health outcomes. While the CGP’s theory of change^1^ (ToC) highlighted how the programme could affect the distribution of power and influence (especially within the households) (Pellerano *et al.*, 2014), the definition and integration of empowerment (especially women’s) into the programme remain unclear.

To inform the study of health inequalities and power issues in CTs like the CGP, the Empowerment for Health Equity—Lesotho (E4HE Lesotho) project used a mixed methods approach to understand the effect of the CGP on the health gap or gradient among young children in the targeted communities, particularly regarding various economic empowerment pathways. This article focuses on how the concept of economic empowerment was perceived by CGP stakeholders in the early phases of the programme.

### Aim and research questions

To address the evidence gaps regarding the role of economic empowerment in CT programmes and its potential contribution to reducing inequalities in targeted communities, this study aims to capture how programme stakeholders understood and operationalized the concept of ‘economic empowerment’—especially women’s—in the early phases of Lesotho’s CGP. Our research questions are as follows:

– How do programme stakeholders define the concept of economic empowerment?
– What role do they see this concept play in the programme?
– Did these roles and definitions evolve over time?
– How do the programme stakeholders perceive the CGP affected economic empowerment in the treatment communities?

### Conceptual background: CT, economic empowerment and health

#### Defining empowerment

The definitions of empowerment are diverse and debated in the literature (Narayan-Parker, 2005a; Luttrell and Quiroz, 2009; Holmes, 2013). To account for that diversity, we adopt a broad definition combining several components in the literature on health or social protection. We define empowerment as either an individual or collective process or an outcome that implies the awareness and capacity to make choices, to act freely on or according to them (agency) to achieve a goal considered desirable (Sen, 1992; WHO, 1998; Kabeer, 1999; Keleher, 2009; Luttrell and Quiroz, 2009; Holmes, 2013; Donald *et al.*, 2020; GEH, 2020). The concept of empowerment is also strongly associated with the feminist movement (Luttrell and Quiroz, 2009), hence the central contribution of women’s empowerment literature to our definition. Women’s empowerment has had growing importance in child health research.

Indicators of women’s social and economic empowerment are associated with improved child health outcomes (Duflo, 2012; Richards *et al.*, 2013; Kuruvilla *et al.*, 2014; Carlson *et al.*, 2015; Cunningham *et al.*, 2015; Taukobong *et al.*, 2016; Thorpe *et al.*, 2016). Women’s economic empowerment is ‘the process by which women acquire access to and control over economic resources, opportunities and markets, enabling them to exercise agency and decision-making power to benefit all areas of their lives’ (Laszlo *et al.*, 2017). Supplementary Annex 1 includes the E4HE Lesotho project’s full conceptual background.

### CTs and empowerment: pathways to impact

At the individual level, CTs can improve recipients’ access to and control over economic resources, their agency and their investment in human development (Barca *et al.*, 2015; UNICEF-ESARO, Transfer Project, 2015; Owusu-Addo *et al.*, 2018). At the household level, CTs can change the household’s socioeconomic conditions as well as individual members’ power and roles, affecting their emotional well-being and intra-household violence or conflicts (Slater and Mphale, 2008; Bastagli *et al.*, 2016; Natali *et al.*, 2018; Barrington *et al.*, 2022). At the community level, CTs can support recipients’ economic, social and political participation and strengthen social cohesion (Barca *et al.*, 2015; Molyneux *et al.*, 2016; Owusu-Addo *et al.*, 2018; de Milliano *et al.*, 2021). Finally, CTs can strengthen the social contract between the State and its citizens and have a transformative impact on power hierarchy and gender norms (Kabeer, 1999; Sabates-Wheeler *et al.*, 2017).

Empowerment has been mainly studied as an outcome of CT programmes for female recipients or members of the household: providing resources to women is thought to benefit children’s health and well-being (Yoong *et al.*, 2012; Richards *et al.*, 2013). However, the evidence remains mixed and highly context-, programme- and outcome-dependent (Bastagli *et al.*, 2016; Bonilla *et al.*, 2017; de Milliano *et al.*, 2021). Hence, to understand the potential impact of the CGP on the empowerment of vulnerable groups (especially women), this study explores the meaning and role this concept plays in the programme from the point of view of those who designed, implemented, funded and evaluated the CGP.

## Method

### Study design

This qualitative case study relied on the triangulation of information from two different sources: desk review (a review of programme documents) and semi-structured interviews with programme stakeholders. Supplementary Annex 2 includes a detailed description of the method.

We focused on the early phases of the programme (2009–13) before the implementation of complementary interventions (Cash Plus). However, elements from the pilot phase (pre-2009) and the post-evaluation phase (post-2014) were considered to understand the evolution of the concepts over time better.

### Study setting

Lesotho has been classified as a least developed country since the establishment of the category (UNCTAD, 2021). Lesotho suffers from recurring political instability fuelled by tensions between political parties, a struggling economy and persistent social and gender inequalities (Shale, 2021). When the CGP started in 2009, more than half of Lesotho children lived in absolute poverty (UNICEF, 2011). The high human immunodeficiency virus (HIV) prevalence rate among adults contributed to rising trends in child mortality and orphanhood (Ministry of Health and Social Welfare - MOHSW/Lesotho, ICF Macro, 2010; UNICEF, 2011; UNFPA, 2012). Despite progress in promoting gender equality in national legislation, customary laws and patriarchal norms had continued to marginalize women and girls, erecting barriers to their access to economic resources and opportunities (UNFPA, 2012; SADC gender protocol 2015; Barometer - Lesotho, 2015).

**Figure 1.**
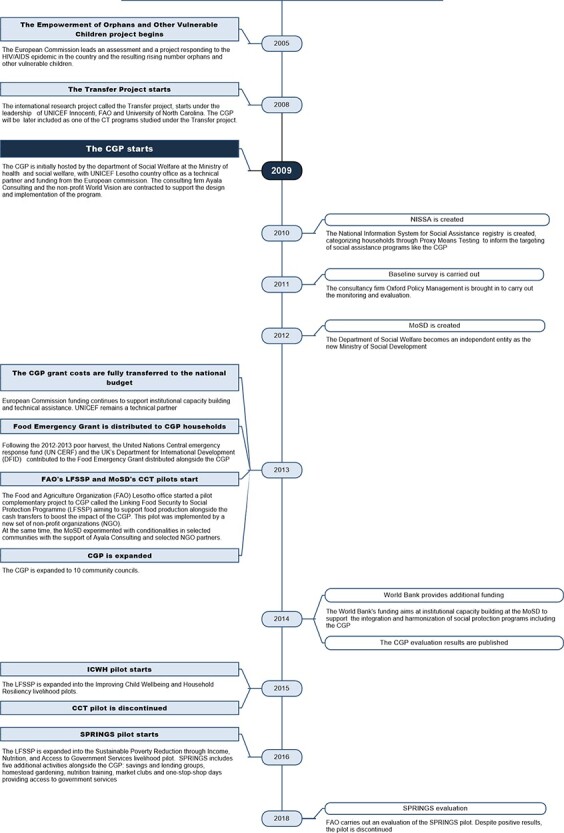
Overview of the CGP between 2005 and 2018 (Pellerano *et al.*, 2014; 2016; UNC Carolina Population Center, UNICEF Office of Research - Innocenti, Food and Agriculture Organization, 2019; Bhalla, 2021)

CT programmes are a key tool in Lesotho’s social protection policy response to these challenges (Granvik, 2016). The CGP was initiated following an assessment from the European Commission (2005–09) responding particularly to the HIV/acquired immune deficiency syndrome (AIDS) epidemic and the resulting rise in OVCs (Pellerano *et al.*, 2016). Figure 1 provides an overview of the CGP (see also Supplementary Annex 2).

### Data collection

To inform our data collection, we mapped CGP stakeholders using programme evaluation documents. To help contextualize the study, we consulted UN agencies in Lesotho involved in the fields of economics, politics, gender, human rights, child health and nutrition at the beginning of the data collection phase.

For the desk review, programme documents were obtained from informants and through a manual search of stakeholder websites carried out between November 2020 and January 2021 (see Table 1).

**Table 1.** Websites searched in the desk review

Website searched	Search dates (month/day/year)	Websites’ home page
Transfer project	11/16/2020 to 11/24/2020	transfer.cpc.unc.edu
UNICEF	12/04/2020	www.unicef.org
UNICEF Innocenti	11/26/2020 to 12/01/2020	www.unicef-irc.org
FAO	11/26/2020 to 12/09/2020	www.fao.org
FAO’s ‘From Protection to Production’ programme	12/04/2020 to 01/06/2021	www.fao.org/economic/ptop
Government of Lesotho’s MoSD	01/06/2021	www.gov.ls/ministry-of-social-development
University of North Carolina at Chapel Hill’s Carolina Population Center	11/28/2020	www.cpc.unc.edu
European Commission	11/16/2020 to 11/20/2020	ec.europa.eu
European Commission’s Delegation to Lesotho	11/18/2020	eeas.europa.eu/delegations/lesotho_en
UK’s Department for International Development (DFID)	11/17/2020	devtracker.fcdo.gov.uk www.gov.uk/government/organisations/foreign-commonwealth-development-office
United Nations Central Emergency Response Fund (UN CERF)	12/01/2020	cerf.un.org
Ayala Consulting Corporation	11/28/2020	ayalaconsulting.us
World Vision	1/06/2021	www.wvi.org
Oxford Policy Management	11/18/2020	www.opml.co.uk
Sechaba Consultants	–	website no longer available as of 28/11/2020
Economic Policy Research Institute	11/18/2020	epri.org.za

Of the 60 documents screened, 51 were included in the analysis (Table 2).

**Table 2.** Number of included programme documents by type

M&E reports	19
Academic papers (produced by programme stakeholders)	12
CGP manuals (e.g. operational manuals and M&E guides)	10
CGP instruments (e.g. survey questionnaires)	3
Stakeholder reports (e.g. annual reports)	3
Internal briefings	3
Press release	1
Total number of documents included	51

For the key informant interviews, the sampling strategy used both purposive and snowballing sampling. Informants had to be either

– professionals directly involved in at least one of the programme cycles of the CGP (strategic development and programme planning, resource mobilization, implementation, monitoring and evaluation (M&E) and/or research) during all or part of the period of interest (even if that person had moved on to a new post) or
– professionals speaking on behalf of the organizations involved in the programme at the time (referred to as ‘Organizational Point of View’).

Twenty-five interviews were conducted between July and August 2021 with informants from UNICEF entities, the Ministry of Social Development (MoSD), the European Commission Delegation in Lesotho, Oxford Policy Management, Food and Agriculture Organization (FAO), World Vision, Ayala Consulting and the World Bank Lesotho. To ensure adequate coverage of the different points of view, informants were further categorized according to the programme cycle, the informants’ role(s) in the CGP (manager, operational staff, analyst/researcher or informant representing the organizational point of view) and whether informants belonged to an international, national or local team or entity. The interviews were conducted online and audio recorded.

We wrote short memos during the desk review and interviews to inform the data collection and analysis.

### Data coding and analysis

We used NVivo 12 for coding and analysis. For the desk review material, the coding was developed deductively using a coding framework based on the conceptual literature (Kabeer, 1999; Graham, 2004; Laszlo *et al.*, 2020), the source and the programme cycle’s phase covered. It was piloted in parallel by two coders and revised before being applied to the whole desk review material. Interview transcripts were coded inductively by one coder, with periodic quality checks by the method specialist. Then, we carried out a thematic analysis. To help identify emerging themes, we used the memos developed during data collection and used the NVivo word frequency function on transcripts and reviewed documents. Disagreements within each theme were explored individually using two-way matrices to identify the determinants of these variations: we reviewed the distribution of points of view across organizations; type of stakeholders (according to role and programme cycle); whether informants belonged to an international, national or local team or entity and the CGP chronology. The documents and interview transcripts were analysed separately, and then we compared their findings.

### Validation

Early findings were discussed with the UNICEF Lesotho country office and the focal point of the MoSD for review and validation.

## Results

### Defining economic empowerment

**Figure 2.**
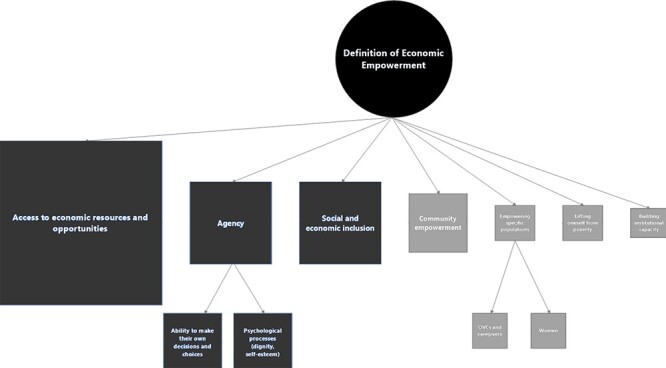
Mapping the definitions of economic empowerment in the CGP

Economic empowerment was defined as a multidimensional concept (Figure 2). We first explore the complexity of this concept across informants before comparing these findings with those of the desk review. We explore the gendered aspects of empowerment separately. 

The size of the boxes is proportional to the number of stakeholders that expressed this view. Dark boxes represent the points of view that achieved consensus, while light boxes indicate areas of disagreement either between stakeholders or between the interviews and the findings from the desk review.

Seventeen informants identified several different dimensions, reflecting the complexity and lack of a unified understanding of this concept (Box 1).

The most common dimension was households’ ‘access to economic resources and opportunities’ (14 interviews). This definition included access to income, assets and markets to generate income and/or improve access to food. A household’s capacity to consume, invest or save was also occasionally mentioned. The second most important dimension of economic empowerment was households’ and individuals’ ‘agency’ (their ability to make their own decisions and choices—seven interviews). When probed further, nine stakeholders associated certain psychological processes with the CGP, such as a sense of dignity, self-esteem and confidence. The third dimension of economic empowerment referred to ‘social and economic inclusion’ in their community, and the fourth dimension mentioned by stakeholders covered the programme’s contribution to households’ and individuals’ ‘lifting themselves from their situation of poverty and vulnerability’ (Box 2).

Box 1. ‘We never gave a proper definition of economic empowerment, so several papers look at the different components of economic empowerment. (…) There was no attempt of defining, in a proper way, the concept of economic empowerment.’

(Researcher/Evaluator, International)

Box 2. ‘Remember, we’re talking about poor people here, who are not able to make any decisions because of their financial status. But if they have something, it also makes them feel like they are part of whatever decisions [that] are being made. They fully participate in the decisions and are able to come up with ideas, economic ideas, that can help them better their lives, make more money and generate income, like participating in this income-generating project that we talked about. For instance, around the councils of [locality], we have, a project that was started by beneficiaries and non-beneficiaries. Because of that, people were able to participate in making sound economic decisions for the betterment of their households.’

(Implementer, Local)

‘When [people involved in strategic discussions] talked about empowerment, ideally you would want people to be lifted out of poverty; people to feel like they can make their own decisions.’

(Implementer, International)

Box 3. ‘I think, within [our organization], we were very strong on building skills and working with the community, to empower the community to make their own decisions. […] It was a lot of community-based trainings and developments, working with the community so that, the community becomes their own answer and their own solution.’

(Implementer, Local)

Finally, five stakeholders—primarily implementers at the local level and international managers—applied economic empowerment to communities from the early phases of the CGP (Box 3). ‘Community empowerment’ referred to building the community’s skills, capacity, inclusivity and ability to generate economic opportunities. Another three stakeholders referred to community empowerment when discussing Cash Plus pilots.

‘Access to economic resources and opportunities, agency and social and economic inclusion’ all were also associated with the concept of empowerment in the desk review (Duynhouwer, 2009; Hurrell *et al.*, 2011; Analysis for Economic Decisions, Analysis for Economic Decisions, 2015; Attah *et al.*, 2016; Ministry of Social Development, Government of the Kingdom of Lesotho and UNICEF, 2016; Pace *et al.*, 2021). Lifting people out of poverty (Barca *et al.*, 2015; Ministry of Social Development, Government of the Kingdom of Lesotho and UNICEF, 2016) was not directly associated with economic empowerment. The documents also highlighted specific populations that CTs like the CGP aim to empower (Cerritelli, 2009; Duynhouwer, 2009; Ayala Consulting, 2010; Thomson and Kardan, 2012; World Vision, 2012; Analysis for Economic Decisions, Analysis for Economic Decisions, 2015): OVCs, their caregivers and women (see Defining gender issues and women’s empowerment). Empowering communities were mainly covered in documents from the more recent phases of the CGP (Ministry of Social Development, Government of the Kingdom of Lesotho and UNICEF, 2016; Pace *et al.*, 2021).

### Defining gender issues and women’s empowerment

**Figure 3.**
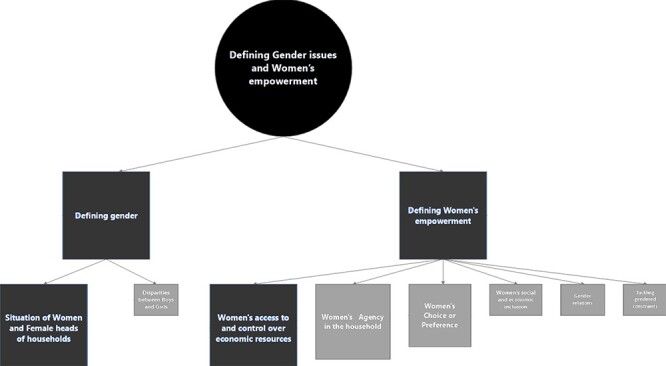
Mapping the definitions of gender issues and women’s empowerment in the CGP

Gender issues and women’s empowerment were mentioned primarily by international organizations, who often equated gender to the situation of women. The definition of women’s empowerment was highly debated (Figure 3). First, we explore the definition of gender in the interviews and the desk review, before discussing the definition of women’s empowerment. 

The size of the boxes is proportional to the number of stakeholders that expressed this view. Dark boxes represent the points of view that achieved consensus, while light boxes indicate areas of disagreement either between stakeholders or between the interviews and the findings from the desk review.

All stakeholders were probed about gender issues in the CGP. All but two stakeholders focused on the disadvantage faced by ‘women’ as compared with men, especially ‘female heads of households’. Only three stakeholders in leadership positions discussed the issue of gender disparities in children, highlighting the disadvantage faced by herd boys (Box 4).

The desk review also revealed a focus on women and especially female-headed households when discussing gender issues in the CGP (Kardan *et al.*, 2011; Hershey, 2012; Pellerano *et al.*, 2012, 2014; World Vision, 2012; Kardan, 2014; Food and Agriculture Organization of the United Nations, 2015; O Campos, 2015; Gavrilovic *et al.*, 2018a; Pace *et al.*, 2019). The focus on gender inequalities amongst children was more present, especially in evaluation reports (UNICEF Lesotho, 2010; Kardan *et al.*, 2011; Pellerano *et al.*, 2012; 2016; Kardan, 2014; Pace *et al.*, 2019).

Looking at women’s empowerment, five stakeholders working for international organizations spontaneously used this term when discussing economic empowerment or gender issues in the CGP.

These stakeholders defined women’s empowerment as giving women ‘access to and control over economic resources and livelihood’, ‘agency’ (decision-making and bargaining power) and ‘choice or preference’ (seen as beneficial to children). They almost systematically linked at least two of these dimensions in their definition, illustrating the multidimensional and debated nature of this concept (Box 5).

Similar dimensions of women’s empowerment were found in the desk review, although agency was more prominent (Hurrell *et al.*, 2011; Kardan *et al.*, 2011; Pellerano *et al.*, 2012; Kardan, 2014; FAO, 2015; O Campos, 2015; Pavanello and Pozarny, 2015). The desk review highlighted further dimensions related to CTs like the CGP: women’s social and economic inclusion in their communities, gender relations and tackling gendered constraints driving women’s disadvantage (e.g. gender norms, access to services or legal rights) (Kardan, 2014; O Campos, 2015; Fisher *et al.*, 2017; Gavrilovic *et al.*, 2018a). Stakeholders discussed these issues (see From theory to practice: role of economic empowerment in the CGP) but did not associate them to the concept of women’s empowerment.

Box 4. ‘Gender was key in Lesotho, because everything we did was around the mother.’

(Implementation Manager, International)

‘One thing which was special in Lesotho is that education is in favour of women because boys are mainly used for herding. They are those who are working with cattle. So many, many boys are missing school.’

(Programme Manager, International)

Box 5. ‘When I say economic empowerment, [I mean that] when women have money in their hands, then they can decide.’

(Programme Manager, International)

‘We’ve been saying for time and time again, that gender-sensitive social protection is very important, high on the priority [list]. However, it’s really difficult to actually define what that actually means.’

(Manager/Organizational Point of View, International)

Box 6. ‘[Economic empowerment] only appears in the policies of the ministry, as a strategic plan of the ministry. But in the operations of this programme or social assistance, no, it doesn’t really come up.’

(Implementer, Local)

‘Economic empowerment was not used [in the CGP] but, in the latter phases of the programme, when we added the Cash Plus component. Then you will find that terminology of economic empowerment.’

(Implementer, International)

### From theory to practice: role of economic empowerment in the CGP

The role and importance of economic empowerment in the CGP were affected by these varying definitions, as they impacted its importance and function in the programme. Fifteen stakeholders addressed the role of ‘economic empowerment’, revealing a clear divide regarding economic empowerment as a CGP objective (Box 6). Evaluation managers and national programme planners saw it as part of the CGP’s focus. Implementers generally agreed that it was not a programme objective in the early phases. This division shows that this concept was part of strategic objectives at the national level but was not translated into operational objectives (five interviews). Later, empowerment became an objective of the various Cash Plus pilots linking CTs and productive or livelihood activities (five interviews).

#### Economic empowerment as access to economic resources and opportunities

**Figure 4.**
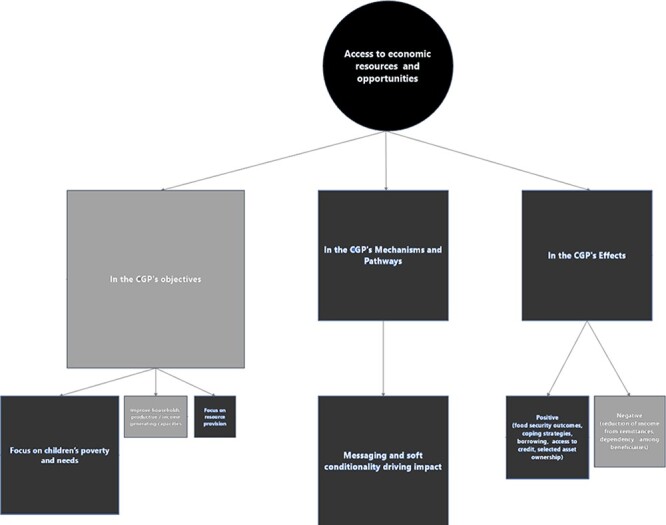
Mapping the role(s) of economic empowerment as access to economic resources and opportunities in the CGP

The role of economic empowerment as ‘access to economic resources and opportunities’ further illustrates the discrepancy between the CGP’s strategic vision and operations (Figure 4) and the evolution of the programme.

The size of the boxes is proportional to the number of stakeholders that expressed this view. Dark boxes represent the points of view that achieved consensus, while light boxes indicate areas of disagreement either between stakeholders or between the interviews and the findings from the desk review.

Twenty-three stakeholders addressed the role of access to resource/opportunity in the CGP, confirming its prominence over the other definitions of economic empowerment. Discussing the programme’s objectives, 10 stakeholders emphasized the focus on children’s (rather than households’) poverty and needs, suggesting that access to economic resources and opportunities was not a prominent objective initially. However, it became more pronounced in the subsequent Cash Plus pilots like SPRINGS (Box 7).

Box 7. ‘The main purpose of CGP programme is to address the poverty of children, not even the poverty of families.’

(Programme Manager, International)

‘CGP is a Cash Plus programme, meaning the people who have the CGP-type of programme, have also access to basic social services or to livelihood means.’

(Programme Manager, International)

Looking at the programme’s design and mechanisms, this focus on children was reflected by the messaging communicated to beneficiary families and their community (soft conditionality—11 interviews). Three stakeholders mentioned that, from the early days of the programme, beneficiaries were told to use the transfer to improve their productive and income-generating capacities. This contradicts statements from other stakeholders and findings from the desk review (see below). Finally, food security was the most common M&E indicator cited by stakeholders, further suggesting that initially, the priority was resource provision rather than economic empowerment (Box 8).

Box 8. ‘It was a wonderful idea to try a holistic approach to all the needs of the family (…) I don’t think they really thought through the process (…) Some households, instead of using the seed [they had received] to plant so that they have food for the coming seasons, [they] ate the seeds. When there’s poverty, when there’s hunger, when there’s famine, I’m obviously going to meet my immediate needs, I’m not thinking about long-term effects or the future.’

(Implementer, Local)

The desk review exposed the gap between the CGP’s ambitions and its operationalization. When describing the objectives and anticipated effects of the CGP, the reviewed documents and ToC included terms related to economic empowerment as access to economic resources and opportunities (Barca *et al.*, 2015; Ministry of Social Development, Government of the Kingdom of Lesotho and UNICEF, 2016). Yet, according to findings from the programme evaluation, beneficiaries and communities were told that investing CGP funds in productive activities was not allowed (Kardan, 2014).

##### CGP affecting access to economic resources and opportunities

The stakeholders’ perception of the CGP’s impact on access to economic resources and opportunities during the early phases of the CGP matched the findings of the desk review. However, stakeholders reported different negative effects than those found in the desk review.

Fifteen stakeholders highlighted the positive impacts of the CGP, primarily on improved consumption and food security outcomes, with some more marginal positive effects on coping strategies, borrowing, access to credit or selected asset ownership (Box 9). As stakeholders often referred to findings from the programme’s evaluation, the positive effects of the CGP documented in the desk review on this dimension of economic empowerment matched those reported in the interviews (Ayala Consulting, 2010; Kardan *et al.*, 2011; Thomson and Kardan, 2012; Kardan, 2014; Pellerano *et al.*, 2014; Dewbre *et al.*, 2015; UNICEF-ESARO, Transfer Project, 2015; Analysis for Economic Decisions, Analysis for Economic Decisions, 2015).

Box 9. ‘I think CGP definitely translated into improving the food security for not only for the children but also for the adults.’

(Resource Mobilization, International)

‘Now [beneficiaries] can actually go out to their neighbour and borrow money. Before then nobody would dare lend you money because they knew you don’t have any.’

(Implementer, International)

‘Most of [the beneficiary households] rely on farming for survival. Some were able to buy farming inputs.’

(Implementer, Local)

Only six stakeholders described negative impacts (Box 10): the size of the transfer being insufficient to trigger empowerment processes; delays in CGP payment affecting beneficiaries’ capacity to improve their livelihood and concerns that beneficiaries may not be prepared to make the necessary efforts leading to economic empowerment.

Box 10. ‘People were meant to be able to rely on this steady source of income and make decisions around that. That certainly wasn’t the case, given these delays and unpredictability.’

(Evaluator, International)

‘Even though they were sensitized to be self-sufficient, to do extra things for the benefit of children or to graduate out of poverty, but the amount is small. So, every time you assess that household, you don’t see a real change. Somehow it just created dependencies like as long as we will be getting money to buy the school uniforms for these children, I think that is fine.’

(Implementation Manager, Local and National)

Box 11. ‘The plan was that if we add the “plus” [interventions] then it would actually complement the cash transfer and would help households improve its economic situation. This programme has been closed now.’

(Implementer, International)

The productive impact of the subsequent livelihood pilots (Cash Plus) was more pronounced (five interviews). However, these were not scaled up, constraining the empowerment potential of the CGP (Box 11).

The desk review showed slightly different findings regarding the negative effects of the CGP. Evaluation documents supported stakeholders’ statements regarding the size of the transfer and the delays in CGP payment (Kardan *et al.*, 2011; Kardan, 2014). However, most of the negative effects described in the evaluation, such as the reduction of income from remittances, were not mentioned by the stakeholders (Kardan, 2014; UNICEF-ESARO, Transfer Project, 2015; Daidone *et al.*, 2017). The desk review also rejected the concerns that the CGP was creating dependency amongst recipients (UNICEF-ESARO, Transfer Project, 2015; Daidone *et al.*, 2017).

#### Economic empowerment as agency

**Figure 5.**
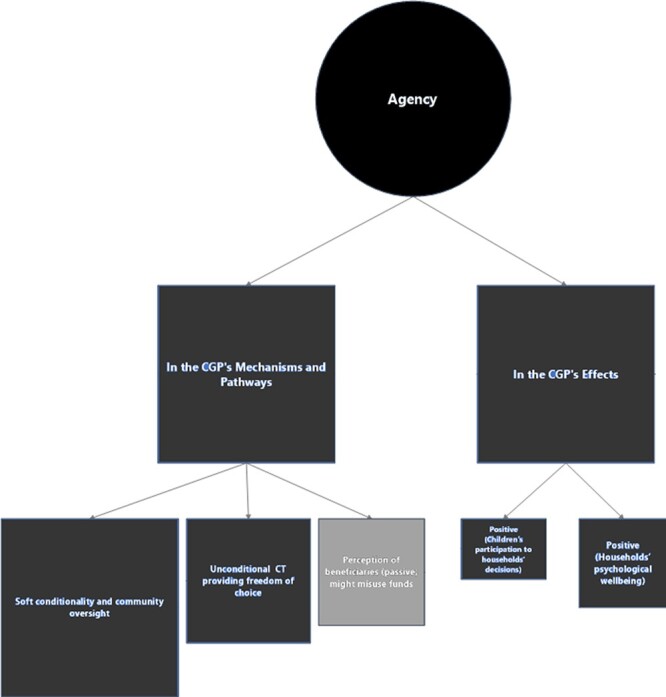
Mapping the role(s) of economic empowerment as agency in the CGP

Empowerment as ‘agency’ appears as a rather minor theme in the CGP’s design (Figure 5).

The size of the boxes is proportional to the number of stakeholders that expressed this view. Dark boxes represent the points of view that achieved consensus, while light boxes indicate areas of disagreement either between stakeholders or between the interviews and the findings from the desk review.

Amongst the 20 stakeholders that discussed beneficiaries’ ‘agency’ in the programme, 17 mentioned it as part of the CGP’s mechanism or effects, particularly regarding conditionality and messaging. Six managers highlighted the empowering potential of an unconditional CT offering more freedom on the use of the funds (Box 12).

This freedom was balanced with other features and mechanisms of the CGP, such as strong community oversight (Box 13): communities ensured that CGP funds were spent on children, steering the spending decisions of the beneficiary households (17 interviews).

The limited empowering capacity of the programme on beneficiaries’ agency was further reflected in how their role was perceived (Box 14). Six stakeholders from national and local teams explained how, in these early phases, beneficiaries’ only role was to comply with the programme’s procedure and objectives. Seven stakeholders also highlighted a prominent concern: beneficiaries misusing the funds.

In the later phases, stakeholders tested two additional mechanisms: conditionality and training activities, which might place additional time and resource burdens onto beneficiaries (Box 15).

As for the desk review, while the programme’s ToC recognizes that the programme may affect intra-household structure and bargaining power, or time preferences, these elements were presented as mechanisms and pathways rather than outcomes the CGP aims to change or influence (Hurrell *et al.*, 2011; Pellerano *et al.*, 2012; 2014). Like stakeholders, programme documents highlighted both the CGP’s empowering potential regarding households’ freedom and decision-making and the constraints linked to soft conditionality and community oversight (Hurrell *et al.*, 2011; Pellerano *et al.*, 2012; 2014; UNICEF-ESARO, Transfer Project, 2015; Pace *et al.*, 2019). How beneficiaries were perceived was less present in the reviewed documents, with the exception of the perceived risk of misusing funds (Cerritelli, 2009; Kardan, 2014; Pellerano *et al.*, 2014; Ayala Consulting, 2014a).

##### CGP affecting agency

The desk review and the interviews agree on the beneficial effect of the CGP in two areas of households’ agency. The previous qualitative evaluation and one implementer highlighted how the CGP improved children’s role in households’ decision-making (Kardan, 2014). Additionally, programme documents and 11 stakeholders described an improvement of beneficiaries’ self-esteem and confidence (Box 16; Pellerano *et al.*, 2014; UNICEF-ESARO, Transfer Project, 2015; Daidone *et al.*, 2017).

Box 12. ‘The grants were meant to give liberty to the parents or the guardians of the children to do anything that, at the end of the day, can benefit the health and welfare of the children, even though there were objectives which were clearly outlined.’

(Implementation Manager, Local and National)

Box 13. ‘The community would not see any significant improvements in terms of consumption, cleanliness and just how the children are still not well taken care of, and some parents would actually be gathering at the chief’s after the collection of the grants. (…) The chief and the [village] committee would try to intervene but if the behaviour of the recipient did not change, transfers would now be collected by someone else deemed more responsible.’

(Programme Manager, Local and National)

Box 14. ‘Some of [the beneficiaries’] were part of these [community] committees. But the great majority of beneficiaries, their role basically was to take care of the children.’

(Implementer, International)

‘There was a lot of preconceived ideas initially, not just from the government, but also from donors in general that cash grants would be used by, especially the man in the family, to smoke or to drink or to use for other purposes.’

(Resource mobilization, International)

#### Economic empowerment as social and economic inclusion

**Figure 6.**
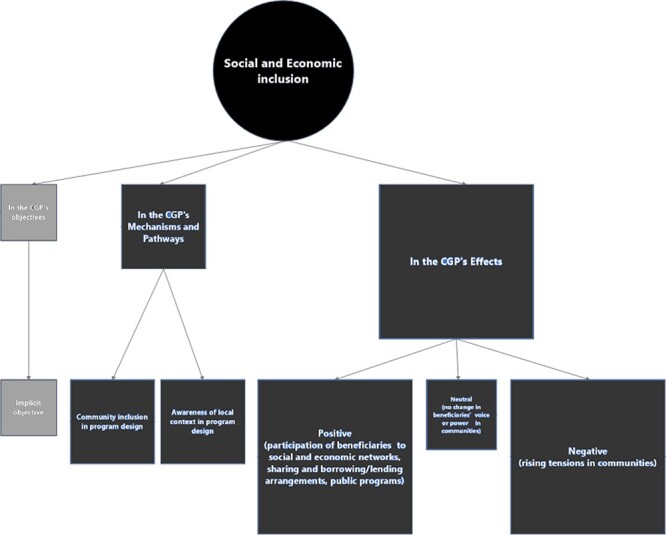
Mapping the role(s) of economic empowerment as social and economic inclusion in the CGP

Economic empowerment as ‘social and economic inclusion’ was considered to be an effect of the CGP, although it was sporadically present in the CGP’s design (Figure 6). 

The size of the boxes is proportional to the number of stakeholders that expressed this view. Dark boxes represent the points of view that achieved consensus, while light boxes indicate areas of disagreement either between stakeholders or between the interviews and the findings from the desk review.

Twenty stakeholders covered the role of this empowerment dimension in the CGP. The re-entry or participation of CGP beneficiaries into the social and economic life and networks of their communities was not a stated objective of the programme in the early phases, although four managers mentioned this as an implicit objective. Inclusion became a clearer CGP objective with the Cash Plus pilot focused on livelihood through community-wide activities (Box 17).

Stakeholders did not consider that the CGP had wider transformative objectives (triggering structural changes that would challenge the socioeconomic or power structure of a community). However, two stakeholders reported certain phases of the programme would consider the communities’ structure and hierarchies (Box 18).

Box 15. ‘While beneficiary households found [the health and nutrition training] to be useful, they were very intense. These households are living not just in monetary poverty, but also time poverty. They do not have a lot of time. To take that time out was challenging because they have to tend to various other tasks as well.’

(Researcher/Evaluator, International)

Box 16. ‘There was a certain level of dignity restored in terms of not only the parents being able to participate in the community as well as the kids being able to participate in all the normal activities that any kid would want to be part of.’

(Programme Manager, Local and National)

Economic empowerment as social and economic inclusion was more present when discussing the CGP’s design. Six stakeholders described how community engagement, beneficiary targeting, case management and the programme evaluation were designed to give a voice to different members of the community (starting with the beneficiaries) and/or safeguard social cohesion (Box 19).

In programme documents, social and economic inclusion or the modification of power structures were not stated as objectives. However, in the ToC, local context and social or economic networks were presented as factors that might boost or hamper the CGP outcomes (Hurrell *et al.*, 2011; Pellerano *et al.*, 2012).

##### CGP affecting social and economic inclusion

The programme documents and 13 stakeholders reported the same positive effects of the CGP: increased participation of CGP beneficiaries in social and economic networks, sharing and borrowing/lending arrangements and public programmes (Box 20; Kardan *et al.*, 2011; Kardan, 2014; Barca *et al.*, 2015; UNICEF-ESARO, Transfer Project, 2015; Attah *et al.*, 2016; Davis *et al.*, 2016). Two managers highlighted greater general acceptance of beneficiaries by their community.

However, implementers working at the community level reported that this increased participation did not translate into beneficiaries having a stronger voice or power in their communities or challenging the community structure (Box 21). This is in line with findings from qualitative evaluations of other CT programmes (Barca *et al.*, 2015; Food and Agriculture Organization of the United Nations, 2015).

Box 17. ‘Even though it was not explicit in the documents, the programme was also meant to facilitate more cohesion in the communities. (…) If there is poverty, it somehow discriminates or divides the community. When we helped these vulnerable households, we are somehow bringing them closer.’

(Implementation Manager, Local and National)

‘With the CGP Plus programme, the idea was [to include] not only the people those who are in [the] CGP programme but also the other people.’

(Programme Manager, International)

Box 18. ‘I don’t think the programme was expected to affect those hierarchies, but I think we were trying to be mindful of those hierarchies when we’re doing an evaluation as part of our explanation of what we see.’

(Evaluator, International)

The desk review and 10 interviews highlighted tensions in the communities resulting from the programme since the first phase of the CGP (Box 21; Cerritelli, 2009; Kardan *et al.*, 2011; Kardan, 2014; Pellerano *et al.*, 2014; Attah *et al.*, 2016). Similarly, programme documents, four implementers and an evaluator referred to concerns over political appropriation of the programme by the community’s leaders (Cerritelli, 2009; Kardan, 2014; Ministry of Social Development, Government of the Kingdom of Lesotho and UNICEF, 2016). These tensions and concerns may counteract other otherwise positive effects of the CGP on empowerment as inclusion.

#### Economic empowerment as lifting oneself from poverty

**Figure 7.**
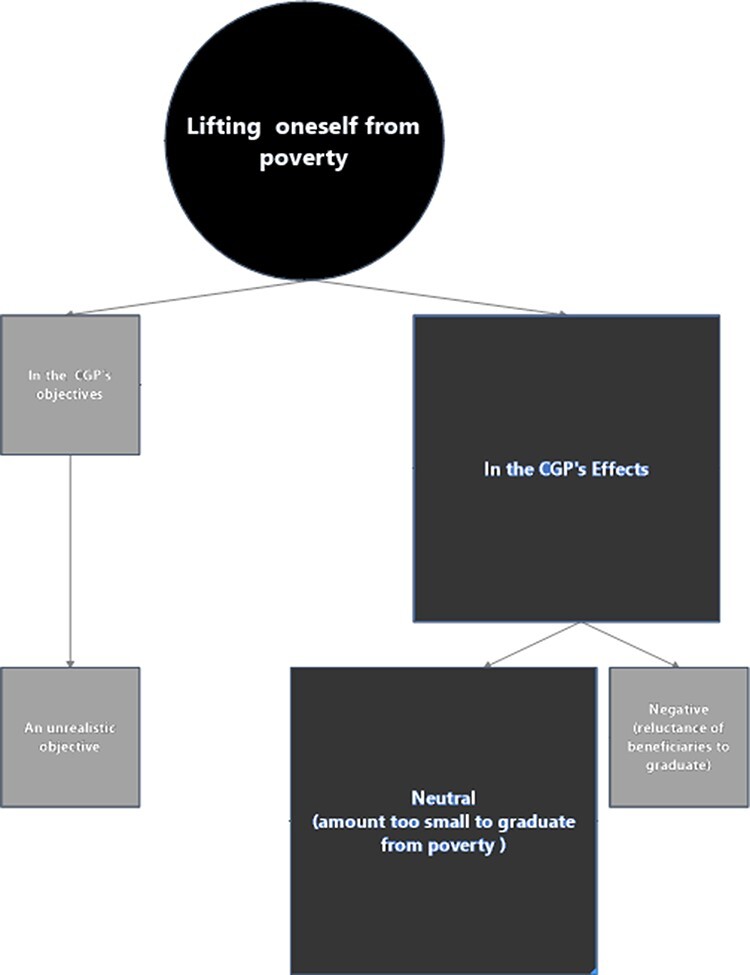
Mapping the role(s) of economic empowerment as lifting oneself from poverty in the CGP

The desk reviews and the interviews with stakeholders tend to disagree on the role of economic empowerment as ‘lifting oneself—or graduation—from poverty’ (Figure 7). 

The size of the boxes is proportional to the number of stakeholders that expressed this view. Dark boxes represent the points of view that achieved consensus, while light boxes indicate areas of disagreement either between stakeholders or between the interviews and the findings from the desk review.

Eighteen stakeholders discussed poverty graduation in our interviews. Five stakeholders considered it an objective of the programme but highlighted the inadequacy of the CT to achieve such an objective. The main reasons for exiting the programme were turning 18 years and leaving the area (six interviews—Box 22).

Box 19. ‘As part of the programme itself, having those various focus groups, I think that gave them a voice, because we tried to reach as many different levels and groups as possible and have representation.’

(Implementer, Local)

Box 20. ‘One of the key outcomes coming from the community is that the CGP has improved [beneficiaries’] status and their profile in the community. Here you can borrow money and tell your neighbour “I will bring it when coming from my collection of the CGP beneficiary funds”. So it is has improved interaction in the community and the community trusts one another in terms of loan.’

(Implementer, International)

‘There were other effects we were not expecting such as [improving] the dignity of families, and the acceptance in a given community.’

(Resource Mobilization, International)

Box 21. ‘I remember reviewing their minutes [of community councils] and looking at who [sits on the council], their decisions and their positionality, (…) I remember coming to a conclusion that the more powerful would have had stronger voices and that even if you had a wider participation, their voices wouldn’t have been strong.’

(Evaluator, International)

‘On the other hand, the programme brought some conflict or tensions, which were unintended, because there is this element of status quo in different communities, that “if I am rich, I should remain rich. If you are poor, you should remain poor”. So, whenever I see some changes in closing the gap—the poverty gap—sometimes it doesn’t sit well with me and increases some elements of conflict because you want to maintain the status quo.’

(Implementation Manager, Local and National)

In the later phases and following the Cash Plus pilots, a graduation model was integrated to the Community Development Model alongside CT programmes like the CGP (Box 23).

None of the programme documents mentioned the graduation objective until the creation of the Community Development Model (Ministry of Social Development, Government of the Kingdom of Lesotho and UNICEF, 2016). Early evaluation and assessment documents stated that there was initially no graduation or exit strategy in place (Ayala Consulting, 2012; Analysis for Economic Decisions, Analysis for Economic Decisions, 2015). Operational documents confirmed that children turning 18 years and the households leaving the intervention area were the main reasons for graduating (Ayala Consulting, 2014b; UNICEF Lesotho, 2017).

##### CGP affecting poverty

Aside from anecdotal cases, the consensus amongst stakeholders was that the CGP was insufficient to lead families to graduate from poverty. This is in line with the findings from a review of CTs in Africa (Barca *et al.*, 2015). Two local implementers also described how some beneficiaries might be reluctant to graduate from the programme, as this would leave them without much needed relief. This was echoed by three managers expressing concerns over the lack of linkage or continuum for the children that had recently left the CGP (Box 24).

#### Economic empowerment as community empowerment

**Figure 8.**
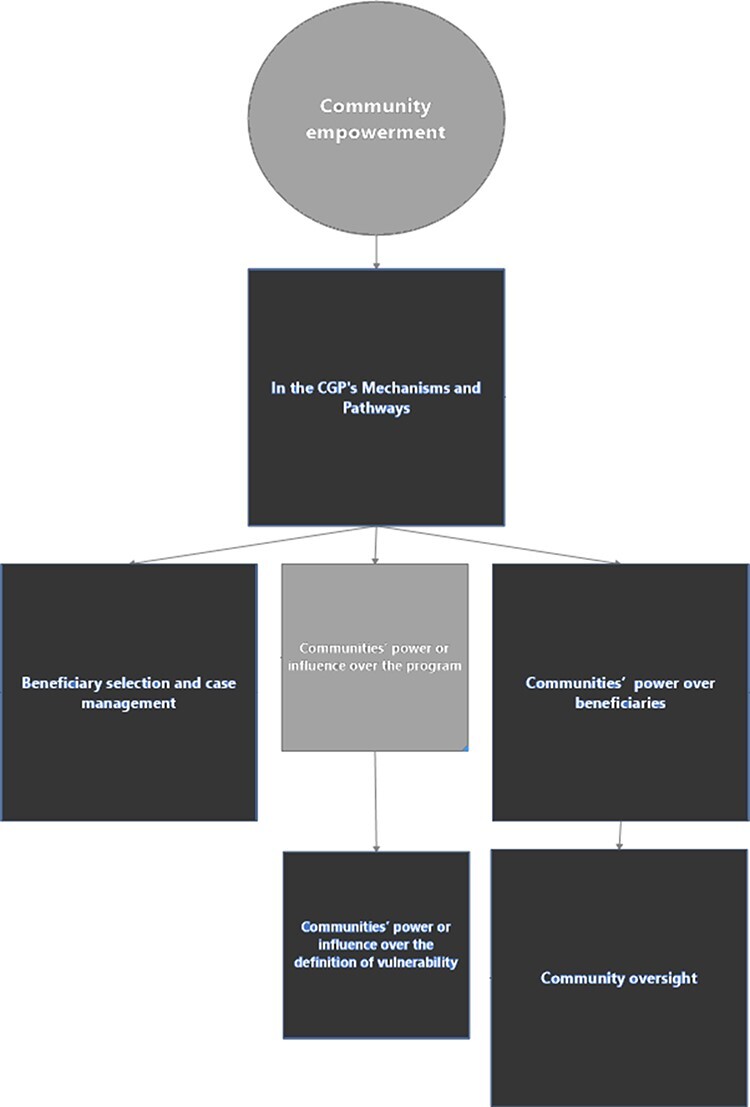
Mapping the role(s) of economic empowerment as community empowerment in the CGP

The role of community empowerment in the CGP was debated (Figure 8). To understand community empowerment in the programme, stakeholders were probed about their interpretation of communities’ definitions and roles (See Supplementary Annex 3).

The size of the boxes is proportional to the number of stakeholders that expressed this view. Dark boxes represent the points of view that achieved consensus, while light boxes indicate areas of disagreement either between stakeholders or between the interviews and the findings from the desk review.

Twenty-two stakeholders described how the community’s role had implications for the empowerment of either communities or beneficiary households. Only five stakeholders mentioned ‘community empowerment’ as a CGP objective from the early phases, but this objective became more prominent in the later phases and pilots (Box 25).

The CGP’s design and implementation were seen as having (dis)empowerment implications for communities. Communities’ power or influence over the programme and its components was unequal. Selected members of the communities were consulted in the initial design of the programme (three interviews). However, those consultations and engagement efforts were not necessarily extensive, except as part of the evaluation (five stakeholders, Box 26). Communities’ involvement in the programme seems to have broadened over time (eight interviews, Box 25).

Communities’ autonomy and influence was more pronounced over the definition of vulnerability (seven interviews) and in the oversight of beneficiaries. Beneficiary selection and/or case management were also identified as local communities’ main roles in the CGP (22 stakeholders, Boxes 27).

Three local implementers also highlighted the communities’ liaison role among the programme, the beneficiaries and institutions (Box 28).

‘Community empowerment’ was widely absent from programme documents, until the SPRINGS pilot and the development of the Community Development Model in the later phases (Ministry of Social Development, Government of the Kingdom of Lesotho and UNICEF, 2016). In the documents, the role of communities primarily involved community participation or programme support in the CGP’s implementation, especially when discussing village committee’s roles (Hurrell *et al.*, 2011; Pellerano *et al.*, 2012; 2014; Kardan, 2014; Ayala Consulting, 2015).

##### CGP affecting community empowerment

Whether the programme’s effect on ‘community empowerment’ was explored in the CGP evaluation and research is unclear: none of the stakeholders interviewed reported on it. It seems that community involvement was used to achieve other aims (Boxes 25–28). This is further confirmed by the desk review. The only community-wide effect reported in the programme documents was the CGP’s economic spillover effect (Kardan, 2014; Pellerano *et al.*, 2014; 2016; Analysis for Economic Decisions, Analysis for Economic Decisions, 2015; Davis *et al.*, 2016).

#### Gender issues and women’s empowerment in the CGP

**Figure 9.**
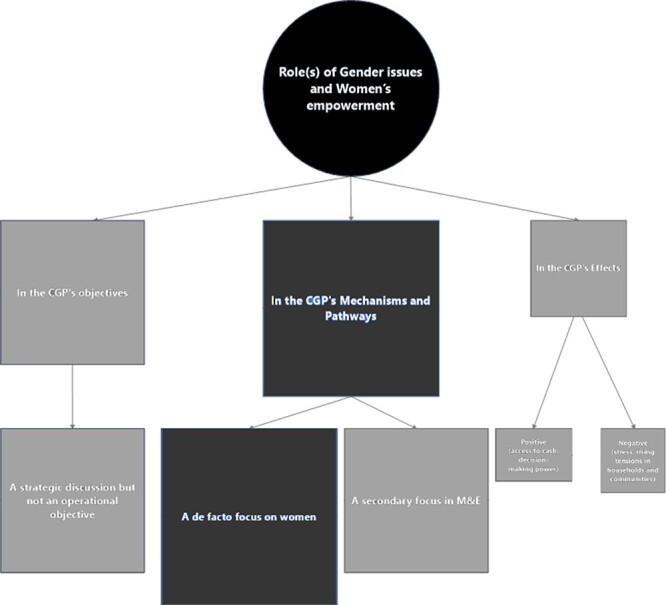
Mapping the role(s) of gender issues and women’s economic empowerment in the CGP

Gender issues and women’s empowerment were not part of the CGP’s formal objectives or design in its early phases, although these may have been part of the strategic discussion (Figure 9). These issues were first formally integrated into the programme as part of the CGP’s evaluation, driven by international organizations.

The size of the boxes is proportional to the number of stakeholders that expressed this view. Dark boxes represent the points of view that achieved consensus, while light boxes indicate areas of disagreement either between stakeholders or between the interviews and the findings from the desk review.

Box 22. ‘At the beginning yes, we were thinking that, to some extent, there was graduation from poverty, but we realized also that by giving only cash, we would not have these results being achieved very quickly.’

(Programme Manager, International)

‘It’s not very common [for a beneficiary to graduate from poverty]. Some graduate because there are no longer kids or because they left the country mostly.’

(Implementer, Local)

Box 23. ‘We have discussed what we call the graduation model. It’s part of the Community Development Department at the ministry. Community Development was instituted, in complement to social assistance, trying to empower households that are benefiting from social assistance, to enable them to graduate.’

(Evaluator, National)

Box 24. ‘Where the programme did not perform well was […] also as far as contribution to poverty was concerned.’

(Implementer, International)

‘The main objective was also to allow them graduate from the programme, but what we realized was that most of them don’t want to graduate. We did a recertification after 5–6 years and most of [the beneficiaries] were left out because people felt their lives had improved, not realizing that they have improved because of the grants that they are getting. And the moment you take them out of the programme, it means you are taking them back to where they were before.’

(Implementer, Local)

Twenty-four stakeholders provided information about the role of gender issues and women’s empowerment. While they were discussed at the strategic level, these issues were not amongst the objectives of the CGP (nine interviews, Box 29).

As for the CGP’s mechanisms and processes, 22 stakeholders reported that gender was not a criterion for targeting CGP beneficiaries nor were there gender-specific features in the implementation of CGP’s early phases. However, these issues became relevant to the CGP as the programme was rolled out due to specificities of the Lesotho context: the vulnerability of female-headed households and women’s role in childcare (10 interviews, Box 30).

Later, the SPRINGS pilot included activities targeting women’s groups specifically to improve their access to economic resources (Box 31).

Only international stakeholders mentioned gender analysis in the context of evaluation (nine interviews), although this primarily consisted of reporting disaggregated data. This would further support the view that gender was a priority specific to international partners further to their organizations’ gender mainstreaming strategies (three interviews, Box 32).

This lack of operational integration of gender and women’s empowerment is reflected in the document review findings: the documents discussed the potential of CTs for women’s empowerment, given the findings from other countries (Kardan *et al.*, 2011; Barca *et al.*, 2015; Food and Agriculture Organization of the United Nations, 2015; FAO, 2015; O Campos and FAO, 2015; Pavanello and Pozarny, 2015; UNICEF-ESARO, Transfer Project, 2015; Fisher *et al.*, 2017; Gavrilovic *et al.*, 2018b). However, none of the CGP operational documents covered the role of these issues in the early phases. Instead, these were covered primarily in M&E reports and related research articles on the CGP’s effects (Kardan *et al.*, 2011; World Vision, 2012; Kardan, 2014; FAO, 2015; O Campos and FAO, 2015; Pace *et al.*, 2019).The CGP evaluations confirmed that women were often the recipients or the decision makers for the CGP funds (Kardan *et al.*, 2011; World Vision, 2012; Kardan, 2014).

##### CGP affecting gender and women’s empowerment

Only a handful of documents and four implementers and evaluators mentioned any impact of the CGP on women’s empowerment. As a result, this remains debated or unknown.

The mechanisms described in both sources of information assumed that, by giving women control over cash, a CT programme like the CGP would improve their position in the household and the community, thus allowing them to re-join community networks and make their own decisions (seen as more child-friendly, Box 33; Kardan *et al.*, 2011; O Campos and FAO, 2015; Pavanello and Pozarny, 2015; Gavrilovic *et al.*, 2018b). Intra-household decision-making and relations were primarily highlighted in the terms of reference for the CGP evaluation (UNICEF Lesotho, 2010).

The reported impact seems nuanced. Three stakeholders said that the CGP was improving women’s access to cash and their power in making decisions for the benefit of the children. Evaluation documents also found improvements in access to resources (including food) in female-headed households (Barca *et al.*, 2015; Pace *et al.*, 2019). However, gender norms and relations within households’ decision-making remained mainly unchanged by the transfer (one interview; Kardan, 2014; FAO, 2015; O Campos and FAO, 2015). At the local level, four implementers described how the transfer might fuel disputes within households, especially when parents were separated. On the contrary, four managers at the national and international levels described how the CGP reduced tensions in some households by reducing the stress due to the lack of income (Box 34). These discrepancies may result from the structure of the household and whether the woman is the sole head of the household.

Finally, at the community level, a previous qualitative evaluation identified that tensions between beneficiary and non-beneficiary households resulting from the CGP were stronger amongst women than men, although the reasons for this were not provided (Kardan, 2014).

## Discussion

This article explored CGP stakeholders’ understanding and operationalization of the concept of economic empowerment in the Lesotho CT programme. We assessed how local, national and international stakeholders involved in different steps of the programme defined this concept, perceived its role(s) and its effects in the CGP. Then, we compared these findings with those from programme documents.

### Main findings

The complexity of economic empowerment found in the literature was reflected in our study (Luttrell and Quiroz, 2009). Both the interviews and the documents acknowledged the multidimensional nature of economic empowerment by including several dimensions in their respective definitions. Some dimensions achieved consensus: access to economic resources and opportunities, agency and social and economic inclusion. ‘Access to economic resources and opportunities’ was the most prominent dimension across the interviews and the desk review. This focus on resources might reflect an approach to social protection still driven by the risk management model rather than by a more transformative approach that further integrates empowerment in CTs (Molyneux *et al.*, 2016). Interestingly, while the reference to Sen’s capabilities approach is widely reflected in the international development literature’s definitions of empowerment (Narayan-Parker, 2005b; Luttrell and Quiroz, 2009; GEH, 2020), this terminology was seldom reflected in some of the stakeholders’ definitions of ‘agency’. Other dimensions were less agreed upon in our study and were primarily used by specific stakeholders: local implementers and international staff tended to highlight economic empowerment as ‘community empowerment’, while ‘graduating from poverty’ was rather used by selected managers.

The diversity of economic empowerment definitions impacted how this concept was integrated into the CGP. The most widely shared definitions of the concept—access to economic resources and opportunities, agency and social and economic inclusion—were also the dimensions with the widest agreement on the role(s) they played in the CGP. The most common definition—‘economic empowerment as improving access to economic resources’—was the most integrated across the programme’s goals, processes and perceived effects on beneficiaries. In comparison, economic empowerment as ‘agency’ was primarily reflected in the CGP’s mechanisms of action, while economic empowerment as ‘social and economic inclusion’ was highlighted as an effect of the programme. There were more disagreements over the role(s) of ‘community empowerment’ or ‘graduation from poverty’. This selective integration of different dimensions of empowerment may result from the dual nature of this concept: a process and an outcome. Luttrell and Quiroz (2009) have illustrated how taking an instrumentalist or a transformative approach to empowerment tends to not only lead stakeholders towards a particular type of interventions but also affect their operational choices in a programme. These findings further illustrate the operational implications of having different understandings of this concept.

Box 25. ‘The second [empowerment dimension in the CGP] was the empowerment of the communities around the aspects of this programme: participating and making sure that [the communities] selected the needy, making sure that they verify attendance, that the families comply with the conditions, that they provide case management and monitoring.’

(Implementation Manager, International)

‘[Community participation] changed with time, but as we are implementing our programmes now, we have taken a lot of lessons from how we started, in terms of implementing the CGP and involving the community towards categorizing people and determining their poverty-ranking statuses.’

(Programme Manager, National)

Box 26. ‘Phase one was “we need to get this project done”. The urgency was so high at that not a lot of time was spent having conversations and engaging with the community.’

(Implementer, Local)

‘Through the qualitative work they would have opportunities to go and ask what the communities perceived about the programme.’

(Evaluator, International)

Box 27. *‘*When we engaged the communities, they also helped us with the local meaning of or local definition of vulnerability, and that is where we also incorporated local knowledge in trying to shape, make or strengthen the targeting mechanism or targeting methods up to this far.’

(Implementation Manager, National and Local)

‘For example, issues like misuse of funds, providing false or fraudulent information were tackled at the community level. If a recipient misuses the funds, their community members would know where to report them and how to handle the cases.’

(Programme Manager, Local and National)

‘This combination of the proxy means testing with a community validation was a way for us to ensure that the community felt respected.’

(Resource Mobilization, International)

Box 28. ‘The committees are linking with the ministry or the auxiliary social worker at the community level, who will be addressing certain issues arising from the recipients.’

(Implementer, Local)

Finally, this study identified gaps in the known effects of the CGP, especially in the more disputed dimensions of economic empowerment such as community empowerment and women’s agency. Although this is unsurprising given the limited and debated operationalization of these dimensions in the programme, the growing focus of Lesotho’s MoSD on these issues as part of the Community Development Model offers new incentives to build consensus on these dimensions’ role and a strong M&E system to assess programme processes and impacts (Ministry of Social Development, Government of the Kingdom of Lesotho and UNICEF, 2016).

Our study reveals three important findings regarding how different stakeholders understood and operationalized economic empowerment in the CGP. First, we observe a gap between the strategic and implementation levels in the operationalization of even the most agreed-upon dimensions of economic empowerment, especially when it came to day-to-day implementation. Feasibility—given the constraints of the Lesotho context (e.g. institutional capacity, funding and local economic context)—may have been a strong contributor to this operationalization gap and appeared to drive some of these decisions.

Second, the division regarding the definition of women’s empowerment and gender issues illustrates a distinction between international and local teams. Women’s empowerment and gender issues were primarily raised by international organizations and stakeholders in leadership positions. This raises the question of whether these concepts were externally imposed. This may also illustrate the wider international debate on the definition and measurement of women’s empowerment (Laszlo *et al.*, 2017; GEH, 2020).

These two key findings address issues about communication and continuity in the programme. The gaps we observed between the strategic and operational levels or among international, national and local teams may be the result of insufficient communication and consensus building between stakeholders. Staff turn-over during and between phases of the programme was not only a challenge for this study but may also have affected communication. While variations in definitions and operationalization of key concepts—if purposeful—may allow more flexibility in a programme to respond to local specificities, we found no indication that this was the case in the early phases of the CGP.

Finally, our study reveals the key role of evaluation in the evolution of stakeholders’ perception of the role of economic empowerment in the CGP. Some dimensions of economic empowerment that were not present in the programme’s early phases (such as social and economic inclusion or community empowerment) became explicit objectives following the first evaluation, with dedicated activities in the Cash Plus pilots. The progressive integration of gender issues and women’s empowerment seemed to have been formally integrated into the CGP primarily through programme evaluation exercises.

### Implication for the CGP and other CT programmes in Africa

This study provides valuable insights into how to strengthen economic empowerment in or through CT programmes in Lesotho and in Sub-Saharan Africa.

Box 29. ‘The underlying goal is to empower people, empower women and close gaps. But it wasn’t in the day-to-day, in the rooms I was sitting in. This wasn’t discussed on a day-to-day basis.’

(Implementer, International)

Box 30. ‘Although there was no specific targeting of women-headed households, I think [it was] because women in themselves are more vulnerable. A lot of men died due to HIV and AIDS, maybe they worked in the mines, they didn’t come back. So, in Lesotho, I think even without going out to say, “we are prioritizing women,” it was evident that women are more in need.’

(Planner, National)

‘It was never mentioned in any programme guidelines that the mothers should be the ones collecting the grants, but in our in our local communities, this time of the day men are out, taking care of their animals, men are out ploughing their fields or harvesting (…) So in gatherings where the government would come and introduce their programmes, it’s sort of a communal norm, [that] a majority of the people who attend those gatherings are the females.’

(Programme Manager, Local and National)

Box 31. ‘Later on, when we introduce the Cash Plus and the Community Development components, we also added some gender activities targeting women empowerment. For example, saving groups [for women].’

(Programme Manager, International)

Box 32. ‘From an M&E and evaluation research perspective, if you have an interest in [women’s] empowerment indicators, it’s often externally imposed, it often comes through the impact evaluations. And that also means that, you know, these indicators are externally imposed as well.’

(Researcher/Organizational Point of View, International)

Box 33. ‘Where the households’ heads are female, the money is actually given to them and then, they have decision-making [power]. Once they have money, they can decide how they will spend the money for children’s education, health…’

(Programme Manager, International)

Box 34. ‘If the families had to separate, they fight for the booklet to receive the child grant.’

(Implementer, Local)

‘Some parents were talking about the possibility of reduced stress, anxiety, or worrying about where the next meal will come from. Because they know that they will always get a certain amount of money they can depend on so that, the parents and the children did not have to worry about money.’

(Programme Manager, Local and National)

Besides clarifying the definitions and role(s) of economic empowerment, this study identified key issues and features of the programme relevant beyond Lesotho. First, this study illustrates how the economic (dis)empowerment of individuals, households, vulnerable groups or communities occurs both out of and through the design of a CT programme. For example, communities’ involvement in undertaking programme tasks was linked, in part, to community empowerment as well as social and economic inclusion. However, the community was also used to oversee beneficiary spending, which might negatively impact beneficiaries’ agency. If the objective of economic empowerment continues gaining prominence in the CGP or in other CTs, it is essential to understand these different processes while agreeing on and prioritizing those deemed most important to the programme.

Second, our study highlights the gaps found between levels of intervention and programme cycles. Although diversity and disagreements are not surprising given the variety and number of stakeholders involved in the CGP as well as the period of time covered, this signals potential issues in the transmission of information between the field and the strategic levels and between the organizations involved. If the path towards a more empowering and gender-sensitive CT programme is pursued further in Sub-Saharan Africa, this points to the importance of discussing and clarifying not only programme objectives but also the meaning of keywords like empowerment.

Finally, the study of gender issues and women’s empowerment in the CGP raises the issue of new objectives being externally brought in without their full adoption and integration into a national programme. Lesotho has committed to mainstreaming gender into its policy and programmes while pursuing a gender-sensitive approach in several areas (Government of the Kingdom of Lesotho, 2018). This study and other research on the CGP have highlighted the relevance of gendered processes within households or communities, which may interfere with the impact of the CGP (Sebastian *et al.*, 2017; Carraro and Ferrone, 2019). This international influence may help raise the profile of such issues in a context where women still face multiple barriers and vulnerabilities (SADC gender protocol 2015 Barometer - Lesotho, 2015; OECD, 2019). However, focusing on women as mothers and female caregivers can reaffirm gender norms, thus limiting the empowering potential of the programme (Nussbaum, 2000; Holmes and Jones, 2010; Molyneux *et al.*, 2016). In the CGP, the way stakeholders described the role of women and the community suggests that the CGP was designed and implemented to conform to existing social and gender norms rather than challenging them. As the Government of Lesotho renews its commitment to reducing gendered barriers (APRM, 2010; Government of the Kingdom of Lesotho, 2018), programmes like the CGP may open new avenues to discuss the role of such programmes in transforming gender norms.

However, conflating ‘gender’ and ‘women’s’ issues risks missing the country’s specificities, such as the increased vulnerabilities of herd boys (Jha and Kelleher, 2006; Lefoka, 2007). This reflects recommendations already formulated in previous research on CTs regarding the importance of understanding empowerment in context in order to properly measure it (Laszlo *et al.*, 2017; Peterman *et al.*, 2021). This study adds to this recommendation by highlighting how understanding empowerment and gender in context can inform the operationalization and integration of these issues into CTs like the CGP. As previous research has stated, many gaps and debates remain in the definition of women’s empowerment as well as in the role this and gender issues play in social protection programmes (Peterman *et al.*, 2019; 2021; Laszlo *et al.*, 2020). In response to these gaps, this study offers a small step towards mapping and understanding how these concepts are perceived and play into complex social protection programmes like the CGP. This is essential to guide stakeholder discussions on the role of gender sensitivity in CTs and better evaluate the impact of such programmes.

### Limitations

Staff turn-over in selected organizations and interview recruitment challenges affected our data collection. When possible, an alternate stakeholder with either organizational knowledge or involvement in part of the phases of interest was identified and interviewed. The desk review was also extensive to try and capture all historical records from programme stakeholders and regularly updated with documents provided by the stakeholders.

The present study focuses on the early phases of the CGP, which started over a decade ago. This may affect the reliability of the information informants recalled. Yet, distance from the period of interest allowed informants to be more reflexive or even critical of these early phases, thus providing a richer, more transparent view of the CGP. To limit the risks of recall biases, informants were asked extensive information about when they were involved in the programme and what their role was over time and were probed about the chronology of certain programme elements. References to specific programme documents or wider events were also used to contextualize the information. When discrepancies were observed in the analysis, we explored whether these might be explained by an evolution of the programme or differences as to when specific informants were involved.

This study focused on the points of view of stakeholders involved in the strategic development and programme planning, resource mobilization, implementation, M&E and/or research of the CGP. In future research, integrating the points of view of recipients and their communities would also enrich the analysis presented here.

## Conclusion

The CGP was initially designed to target the multidimensional vulnerabilities affecting children in a context of widespread poverty, food insecurity and the HIV/AIDS epidemic. Our study found that the initial ambitious vision of this programme at the strategic level explicitly or implicitly touched upon many dimensions of economic empowerment and gender. We identified five key dimensions of economic empowerment: access to economic resources and opportunities, agency, social and economic inclusion, community empowerment and lifting families out of poverty (or graduation). Women’s empowerment tended to echo several of these dimensions. Gender was overwhelmingly used to refer to the situation of women (or female heads of households specifically). Most sources and informants identified more than one dimension in their definitions of these concepts, thus illustrating their complexity as applied to the CGP. Economic empowerment as ‘access to economic resources and opportunities’ (for all beneficiaries or for women in particular) was the most prominent and integrated dimension of economic empowerment across the programme. However, our study found discrepancies and disagreements in the operationalization of these concepts, affecting the least agreed-upon definitions in particular. Second, apart from access to resources, all other dimensions of these concepts were operationalized selectively throughout the programme. This uneven operationalization has highlighted how different empowerment processes may conflict with one another, thus affecting the impacts of the programme. However, these discrepancies have also highlighted more systematic divisions, particularly between the strategic and operational levels of the programme—pointing to operationalization gaps as well as stakeholder-specific agendas and priorities. Because of their debated role(s) and importance in the CGP, several potential effects of the CGP, such as its community empowerment and women’s empowerment effects, remain understudied.

## Supplementary Material

czad009_Supp

## Data Availability

The data underlying this article cannot be shared publicly to protect the anonymity of the respondents who participated in the study.

## Footnotes

1. The ToC is a methodology designed to support strategic planning and evaluation in development programs. It can be used as a tool to lay out the logical sequence of a program to describe how individual activities might lead to the desired change or outcomes and the different pathways through which this change may occur (Vogel, 2012).

## Abbreviations

CGP = Child Grants Programme

CT = cash transfer

DFID = Department for International Development

FAO = Food and Agriculture Organization

ICWH = Improving Child Wellbeing and Household Resiliency

LFSSP = Linking Food Security to Social Protection Programmeme

LMICs = low- and middle-income countries

M&E = monitoring and evaluation

MoSD = Ministry of Social Development

NISSA = National Information System for Social Assistance

OCVs = orphans and vulnerable children

SPRINGS = Sustainable Poverty Reduction through Income, Nutrition and Access to Government Services

ToC = theory of change

UN CERF = United Nations Central Emergency Response Fund

UNCTAD = United Nations Conference on Trade and Development

VAC = Village Assistance Committee

WEE = women’s economic empowerment

## Reflexivity statement

To ensure the relevance of this research to policymakers in Lesotho, the collaboration behind this article included both national and international researchers and practitioners, selected because of their expertise on Lesotho, cash transfers and/or methodology. The authors’ group includes Europeans, American and African nationals, based in Norway and in Lesotho. This article was led by early-career researchers and practitioners, with support from more experienced professionals. The authors also ensured a review and validation of findings through an internal validation process involving civil servants in charge of the programme of interest, international organizations present in Lesotho and academics with expertise in cash transfers.
