# Supplementary material for: Exploring economic empowerment and gender issues in Lesotho’s Child Grants Programme: a qualitative study
Source: Health Policy Plan. 2023 Feb 8;39(2):95–117. doi: 10.1093/heapol/czad009 (PMC11651286; doi:10.1093/heapol/czad009)
Supplement: czad009_Supp [file czad009_supp.zip › E4HE 1 Empw Annex 2Rev1Clean.docx]

Annex 2. Method

Study design

This qualitative case study relied on the triangulation of information from two different sources: desk review - a review of program documents (e.g. briefs, analyses and reports generated by program stakeholders) and semi-structured interviews with program stakeholders.

This study primarily focuses on the early phases of the program (2009-2013), prior to the implementation of complementary interventions (Cash Plus). However, elements and consideration from the pilot phase (pre-2009) and the post-evaluation phase (post-2014) were also considered to better understand the evolution of the concepts overtime.

Study setting

Lesotho has been classified as a least developed country by the United Nations Conference on Trade and Development (UNCTAD) since the establishment of the category (UNCTAD, 2021). When the CGP started in 2009, more than half of Lesotho children lived in absolute poverty (i.e. they were deprived in two or more essential dimensions), with rates ranging from 31% in the lowlands to more than 80% in the mountain areas.(UNICEF, 2011) The HIV prevalence rate amongst adult was 23%, the third highest rate globally, contributing to rising trends in maternal and child mortality between 2004 and 2009 (Ministry of Health and Social Welfare - MOHSW/Lesotho and ICF Macro, 2010; UNICEF, 2011; UNFPA, 2012). The HIV burden disproportionally affect women (Ministry of Health and Social Welfare - MOHSW/Lesotho and ICF Macro, 2010). Despite progress in promoting gender equality in national legislation, customary laws and patriarchal norms had continued to marginalize women and girls and erect barriers to their access to economic resources and opportunities (UNFPA, 2012; *SADC gender protocol 2015 Barometer - Lesotho*, 2015). Although Basotho women tend to be better educated than their male counterparts, they continued to face lower levels of employment and lower wages (Ministry of Health and Social Welfare - MOHSW/Lesotho and ICF Macro, 2010; OECD, 2019). Domestic and gender-based violence have remained widespread (Ministry of Health and Social Welfare - MOHSW/Lesotho and ICF Macro, 2010; UN Lesotho, 2021). Lesotho had suffered from recurring political instability, fueled by tensions between political parties, a struggling economy and persistent social and gender inequalities (Shale, 2021). For example, since the CGP started, Lesotho faced a series of three elections between 2012 and 2017, and in 2014, violent incidents broke out between the army and the policy further to an alleged attempted coup (Shale, 2021). Economically, Lesotho and its workforce had remained heavily dependent on South Africa. Hence, South Africa’s economic crisis in the 1990s and the retrenchment of thousands of Basotho mine workers had long term impacts on households’ incomes, especially in rural areas (UNICEF, 2011; Granvik and UNU-WIDER, 2016; Ministry of Labour and Employment and IOM, 2017). Finally, Lesotho has suffered from regular extreme weather events, leading to food insecurity (Shale, 2021). For example, flooding in 2010-2011 followed by a prolonged drought the following year heavily impacted food security in rural Lesotho, including the areas where the CGP was rolled out (Pellerano *et al.*, 2014).

In response to these challenges, Lesotho has made growing commitments to the development of a strong social protection system (UNICEF Lesotho, 2020). Cash transfers programs are a key tool in this system and are core social assistance and poverty reduction instruments, particularly for children (Granvik and UNU-WIDER, 2016; UNICEF Lesotho, 2020). When the Ministry of Social Development (MoSD) was created in 2012, over 400 million maloti (LSL) (about 2.5% of Lesotho’s GDP) were spent on the three main governmental CT programs (Table 1), one of the highest spending level in the region (Smith *et al.*, 2013).

*Table 1. Overview of CT programs in Lesotho (as of 2012) (Smith et al., 2013)*

| **CT program** | **Target population** | **Benefits** | **Number of beneficiaries** | **Annual expenditure** | **Started in** | **Leading governmental institution** |
| --- | --- | --- | --- | --- | --- | --- |
| **Public assistance** | “Destitute” (extremely poor individual) | In-kind or cash transfers of an average of LSL 100 /month /person | 9 500 individuals | LSL 13 million  Source: Government funding | 1970’s | MoSD* |
| **Old Age Pension** | All Basotho over 70 years old (excluding civil servant pensioners) | Cash transfers of LSL 350 /month /person | 84 000 individuals | LSL 371 million  Source: Government funding | 2004 | Ministry of Finance |
| **Child Grants Program** | Poor and vulnerable households with children | Cash transfers of LSL 360 /quarter /household (later modulated from LSL 300 to 750 according to the number of children) | 10 000 households | LSL 16,5 million  Source : European Commission | 2009 | MoSD* |

*Previously under the Department of Social Welfare in the Ministry of Health and Social Welfare

Unlike other CTs in Lesotho, the CGP was distributed quarterly and targeted households rather than individuals (Smith *et al.*, 2013). From about 1,250 households in three Community Councils in the districts of Qacha’s Nek, Mafeteng and Maseru when the program started, the CGP reached 2,300 households in ten Community Councils in the five districts of Qacha’s Nek, Maseru, Leribe, Berea and Mafeteng at the time of the first evaluation in 2013. Beneficiaries mainly live in rural communities in the lowlands and foothills, with limited access to services and markets. Households were selected based on a community census through Proxy Means Testing^^[[1]](#footnote-1)^^, which identify ultra-poor and very poor households. Local communities were also involved in the identification and selection of poor and vulnerable households eligible for the CGP through a process of community validation, carried out alongside the census-based selection.^^[[2]](#footnote-2)^^ The CGP was initiated following an assessment from the European Commission (2005-2009), in response to the HIV/AIDS epidemic in the country and the resulting rising number of OVCs (Pellerano *et al.*, 2016). In the early phases, the program was designed as a randomized controlled trial, where electoral divisions in the Community Councils part of the CGP were randomly assigned to a treatment group (where the CGP was implemented) and a control group (where eligible households were identified but the implementation was postponed until after the evaluation). Although designed as an unconditional^^[[3]](#footnote-3)^^ CT, strong messaging or soft conditionality were implemented: CGP beneficiaries were instructed at pay points and in their community that the transfer was to be used for the children (Pellerano *et al.*, 2014).

Figure 1 (in the main document) provides an overview of the CGP’s timeline and the organizations involved.

Data collection

To inform our data collection, we carried out an initial mapping of CGP stakeholders using program evaluation documents. This initial mapping was updated iteratively according to the findings of the desk review and contacts provided by key informants.

To help contextualize the study, the research team consulted UN agencies in Lesotho involved in the fields of economics, politics, gender, human rights, child health and nutrition at the beginning of the data collection phase.

For the desk review, program documents were primarily obtained through a manual search of stakeholder websites (as identified in the stakeholder mapping) and associated program’s pages. These included the Transfer project, UNICEF, UNICEF Innocenti, FAO and its “From Protection to Production” project pages, the Government of Lesotho’s Ministry of Social Development, the University of North Carolina at Chapel Hill’s Carolina Population Center, the European Commission and its Delegation to Lesotho, the UK’s Department for International Development (DFID), United Nations Central Emergency Response Fund (UN CERF), Ayala Consulting, World Vision, Oxford Policy Management, Sechaba Consultants, and Economic Policy Research Institute (see Annex 1 for links to webpages). This search was carried out between November 2020 and January 2021. Of the 60 documents screened, 51 were included in the analysis. These include 19 monitoring and evaluation (M&E) reports, 12 academic papers produced by program stakeholders, 10 manuals designed to guide theifferrent stage of the program cycle (e.g. operational manuals, M&E guides), three program instruments (e.g. survey questionnaires), three stakeholder reports describing their contribution and activities in the CGP (e.g. annual reports), three internal briefing and one press release.

For the key informant interviews, the sampling strategy used both purposive and snowballing sampling. A list of stakeholders and potential contacts was developed by the lead researcher using the initial stakeholder mapping and reviewed by [the authors institution]. To be included, informants had to be either

1) A person or organization that was involved in the strategic development and program planning, resource mobilization, implementation, monitoring and evaluation and/or research of the CGP during all or part of the time period of interest (even if that person had moved on to a new post)

2) or a person who can speak on behalf of the organization involved in these program phases (spokesperson, referred to as “Organizational Point of View”)

Professionals representing organizations that became involved in the CGP after the period of interest were excluded. We aimed to include at least one person per role in the organization (manager, operational staff, analyst/researcher or informant representing the organizational point of view), and program cycle (strategy development and program planning, resource mobilization, implementation, monitoring and evaluation (M&E) and/or research).

As the interviews progressed, the stakeholder list was reviewed and when relevant, updated with the contacts provided by the key informants themselves. Potential informants were contacted by email or phone with facilitation from [the authors institution].

Twenty-five interviews were conducted between July and August 2021 with informants from UNICEF entities, the MoSD, the European Commission Delegation in Lesotho, Oxford Policy Management, FAO, World Vision, Ayala consulting, and the World Bank Lesotho. To ensure adequate coverage of the different points of view, informants were further categorized according to the program, the informants’ role(s) in the CGP and whether informants belonged to an international, national or local team or entity.

Due to period covered and staff turnover in some of the targeted organizations, certain roles were covered by two different informants. Nine informants also changed roles and/or organizations while continuing to work on the CGP. When a target informant was unavailable, or unwilling to take part in this research, the team attempted to secure the participation of an alternate. However, we were unable to reach or secure interviews with five of our target informants.

An interview guide was developed by the project leader and method specialist with the support of [the authors institution] for each type of stakeholder according to their role in the program. The interviews were conducted online by the project leader and the research assistant or the research liaison, and audio recorded. Each interview was transcribed and anonymized by the project leader and the research assistant. A code was assigned to each interviewee to ensure anonymity.

To help explore potential conflicts or new themes, the interviewers wrote short memos after each interview and as they reviewed program documents. These memos informed the evolution of the interview guide and the prioritization of questions with individual stakeholders.

Data coding and analysis

The documents collected through desk review and the transcripts from the interviews were coded using NVivo 12. For the desk review material, the coding was developed deductively, based on an initial coding framework developed according to the conceptual literature (Kabeer, 1999; Graham, 2004; Laszlo *et al.*, 2020), the source, and the program cycle’s phase covered. This coding system was piloted with a first batch of five documents, coded in parallel by the lead researcher and the research assistant to test the suitability of the coding book and inter-rater reliability between coders. The coding system was revised accordingly. Discrepancies in coding were discussed between coders and solved by consensus. The rest of the documents were coded by one coder, with a sample cross-checked by the other coder. Regular meetings between coders were held to discuss and solve differences and challenges in document coding. Throughout coding, team members kept a memo on their questions, thoughts and reflections to inform the interviews and the analysis.

Interview transcripts were coded inductively. All the transcripts were coded by one coder, with periodic quality check by the method specialist, to ensure consistent information capture and coding application. Codes were grouped into pre-defined categories similar to those used for the document coding. However, the framework was kept flexible to allow new categories arising from interviews. During coding, team members wrote notes and memos to start identifying patterns and themes.

To analyze the data, the documents and interview transcripts were initially categorized by organization and type of stakeholders (according to role and program cycle). However, as initial data exploration and the memos written throughout the data collection showed, this categorization was found inadequate to explore the different understanding and operationalization revealed by the data. Instead, a thematic analysis was used. To help identify emerging themes, the team used the memos developed during data collection and coding, as well as the NVivo word frequency function on individual transcripts or coded sections in the reviewed documents. To ensure an adequate contextualization of the data, potential interactions between context codes and each code category (e.g. definitions, roles or effects) were explored using a two-way matrix. Differences or disagreements found within each theme were explored individually, to find if a determining factor for these variations could be identified. To do so, the team used two-way matrices to explore the distribution of points of views across organizations; type of stakeholders (according to role and program cycle); whether informants belonged to an international, national or local team or entity; and the period of the CGP the informants or documents covered. The documents and interview transcripts were analyzed separately, then the findings were compared for each theme.

Validation

Early findings were presented and discussed with [the authors institution] and the focal point of the MoSD for review and validation.

## References

Graham H. 2004. Tackling Inequalities in Health in England: Remedying Health Disadvantages, Narrowing Health Gaps or Reducing Health Gradients? *Journal of Social Policy* **33**: 115–31.

Granvik M, UNU-WIDER. 2016. *Policy diffusion, domestic politics and social assistance in Lesotho, 1998–2012*. UNU-WIDER.

Kabeer N. 1999. Resources, Agency, Achievements: Reflections on the Measurement of Women’s Empowerment. *Development and Change* **30**: 435–64.

Laszlo S, Grantham K, Oskay E, Zhang T. 2020. Grappling with the challenges of measuring women’s economic empowerment in intrahousehold settings. *World Development* **132**: 104959.

Ministry of Health and Social Welfare - MOHSW/Lesotho, ICF Macro. 2010. Lesotho Demographic and Health Survey 2009. MOHSW and ICF Macro., Maseru.

Ministry of Labour and Employment, IOM. 2017. Labour Migration Assessment: Lesotho. IOM.

OECD. 2019. Social Institutions and Gender Index - Lesotho profile. OECD, Paris.

Pellerano L, Daidone S, Davis B, *et al.* 2016. Does Evidence Matter? Role of the Evaluation of the Child Grants Programme in the Consolidation of the Social Protection Sector in Lesotho. In: Davis B (ed). *From evidence to action: the story of cash transfers and impact evaluation in Sub-Saharan Africa*. First edition. Oxford University Press ; Food and Agriculture Organization of the United Nations ; United Nations Children’s Fund: Oxford : [Rome, Italy] : New York, NY, 247–80.

Pellerano L, Moratti M, Jakobsen M, Bajgar M, Barca V. 2014. The Lesotho Child Grants Programme Impact Evaluation: Follow-up Report. UNICEFLesotho (with EU funding and technical support from FAO), Maseru.

SADC gender protocol 2015 Barometer - Lesotho. 2015. Gender links, Johannesburg, South Africa.

Shale V. 2021. Understanding Conflict, Peace and Gender Context in Lesotho. Government of the Kingdom of Lesotho; UNDP; UN agencies in Lesotho, Maseru.

Smith WJ, Mistiaen E, Guven M, Morojele M. 2013. Lesotho - A Safety Net to End Extreme Poverty

UN Lesotho. 2021. Lesotho Common Country Analysis | United Nations in Lesotho. UN Lesotho, Maseru.

UNCTAD (ed). 2021. *The least developed countries in the post-COVID world: learning from 50 years of experience*. United Nations Publications: New York.

UNFPA. 2012. UNFPA Country programme document for Lesotho 2013-2017.

UNICEF. 2011. Child Poverty in Lesotho.

UNICEF Lesotho. 2020. UNICEF Lesotho 2020/21 Social Protection Budget Brief.

1. The Proxy Means Testing is a method designed assess the household’s level of poverty. In Lesotho, this assessment was based on dwelling conditions, households characteristics and the ownership of selective assets (Pellerano *et al.*, 2014). [↑](#footnote-ref-1)
2. The role and importance of communities in the CGP beneficiary selection process as well as the methodology used evolved over time. [↑](#footnote-ref-2)
3. Unlike conditional CTs, **un**conditional CTs do not require that beneficiaries meet specific milestones or adopt specific behavior (e.g. child vaccination, school enrollment, training sessions) to keep receiving the transfers. [↑](#footnote-ref-3)
