# Supplementary material for: Exploring economic empowerment and gender issues in Lesotho’s Child Grants Programme: a qualitative study
Source: Health Policy Plan. 2023 Feb 8;39(2):95–117. doi: 10.1093/heapol/czad009 (PMC11651286; doi:10.1093/heapol/czad009)
Supplement: czad009_Supp [file czad009_supp.zip › E4HE 1 Empw Annex 3Rev1clean.docx]

# Annex 3.

Understanding the concept of community in the CGP

Of the 11 stakeholders who provided a definition of “the community” in the context of the CGP, eight referred to the CGP’s community committee (the Village Assistance Committee - VAC). It is unclear whether any member of the community had a chance to be part of the VAC, as four stakeholders mainly identified village officials as VAC members (Box 1). The involvement of beneficiaries themselves into these committees was only mentioned by two stakeholders.

**Box 1**

| “VACs were composed of health workers and sometimes the teacher or the chief... like, eminent people in the village.”  (Resource mobilization, International) |
| --- |

International stakeholders also highlighted how the VACs were sometimes used to exercise political influence (Box 2).

**Box 2**

| “The committee was hijacked by few influential people who were part of the committee, like the chief and or the counselor. And therefore, the VAC, to some extent, would bring their people, people who are politically inclined to them. So the communities really complained.”  (Implementer, International) |
| --- |

Over time, the CGP moved away from this approach to better involve the community at large (Box 3).

**Box 3**

| “In the current methodology, the community is everybody who is an adult. It’s open community-based targeting [of CGP recipients]. In the previous methodology, it used to be a selected committee that would do that, but we had challenges with that kind of approach and now we have moved to this one, that is open for all community members”  (Implementer, International) |
| --- |

The desk review shows a wider definition of community than that of the stakeholders’ but also, a distinction between the VAC and the “community” at large – the VAC being primarily described as an operational entity of the CGP (Pellerano *et al.*, 2012, 2014). Some of the evaluation documents highlighted that VACs were not well integrated into local community and government processes, and raised questions as to whether the VAC was truly representative of the community (Thomson and Kardan, 2012; Oxford Policy Management *et al.*, 2014; Bhalla, 2021). As for its composition, program documents described the VAC as including both community officials and “respected members of the community” (Pellerano *et al.*, 2012), although the concerns over the VAC being used for political influence was also found in the program’s evaluation (Pellerano *et al.*, 2012).

In the documents, the role of communities primarily aimed at either community participation or program support in the CGP’s implementation (especially when discussing VAC’s roles), particularly around beneficiary selection, case management and oversight (Hurrell *et al.*, 2011; Pellerano *et al.*, 2012, 2014; Oxford Policy Management *et al.*, 2014; Ayala consulting, 2015). These main roles are similar to those highlighted in the stakeholder interviews. In contrast, the role of communities in the development and planning of the program was widely absent in the reviewed documents.

Second, evaluation documents raised questions as to whether communities’ roles in the program were effectively implemented – an issue that was widely absent from the informants’ perspective. First, the qualitative evaluation found a gap between the role of the VAC as it was planned in program documents and their actual role on the ground, which appeared more limited (Oxford Policy Management *et al.*, 2014). Second, several evaluation documents found that the role of the VAC in beneficiary selection, while effectively implemented, wasn’t initially visible to communities to avoid tensions and retaliations against VAC members (Pellerano *et al.*, 2012, 2014; Thomson and Kardan, 2012).

# References

Ayala consulting. 2015. Technical assistance to the government of Lesotho for capacity building, skills transfers, scale up and transitional arrangements under the Lesotho Child Grants Programme CGP Final report.

Bhalla G. 2021. Institutional assessment of the Child Grants Programme and Sustainable Poverty Reduction through Income, Nutrition, and Access to Government Services pilot project in Lesotho. FAO, Rome, Italy.

Hurrell A, Pellerano L, MacAuslan I, Merttens F, Kardan A, Oxford Policy Management. 2011. CGP impact evaluation - Inception report. Oxford Policy Management, Oxford.

Oxford Policy Management, Andrew Kardan, FAO. 2014. Qualitative Research and Analyses of the Economic Impact of Cash Transfer Programmes in sub-Saharan Africa. Lesotho Country Case Study Report. From Protection to Production project report. FAO, Rome, Italy.

Pellerano L, Hurrell A, Kardan A, *et al.* 2012. CGP Impact Evaluation. Targeting and Baseline Evaluation Report’. Report prepared for the Government of Lesotho. Oxford Policy Management, Maseru.

Pellerano L, Moratti M, Jakobsen M, Bajgar M, Barca V. 2014. The Lesotho Child Grants Programme Impact Evaluation: Follow-up Report. UNICEFLesotho (with EU funding and technical support from FAO), Maseru.

Thomson A, Kardan A. 2012. Final Evaluation of Support to Lesotho HIV and AIDS Response: Empowerment of Orphans and Other Vulnerable Children. Oxford Policy Management, Oxford.
